# Are citizen juries and assemblies on climate change driving democratic climate policymaking? An exploration of two case studies in the UK

**DOI:** 10.1007/s10584-021-03218-6

**Published:** 2021-09-16

**Authors:** Rebecca Wells, Candice Howarth, Lina I. Brand-Correa

**Affiliations:** 1 Grantham Research Institute on Climate Change, London School of Economics and Political Sciences, London, UK; 2 Sustainability Research Institute, University of Leeds, Leeds, UK

**Keywords:** Climate policy, Democratic deliberative process, Citizen jury, Citizen assembly

## Abstract

In light of increasing pressure to deliver climate action targets and the growing role of citizens in raising the importance of the issue, deliberative democratic processes (e.g. citizen juries and citizen assemblies) on climate change are increasingly being used to provide a voice to citizens in climate change decision-making. Through a comparative case study of two processes that ran in the UK in 2019 (the Leeds Climate Change Citizens’ Jury and the Oxford Citizens’ Assembly on Climate Change), this paper investigates how far citizen assemblies and juries are increasing citizen engagement on climate change and creating more citizen-centred climate policymaking. Interviews were conducted with policymakers, councillors, professional facilitators and others involved in running these processes to assess motivations for conducting these, their structure and the impact and influence they had. The findings suggest the impact of these processes is not uniform: they have an indirect impact on policy making by creating momentum around climate action and supporting the introduction of pre-planned or pre-existing policies rather than a direct impact by truly being citizen-centred policy making processes or conducive to new climate policy. We conclude with reflections on how these processes give elected representatives a public mandate on climate change, that they help to identify more nuanced and in-depth public opinions in a fair and informed way, yet it can be challenging to embed citizen juries and assemblies in wider democratic processes.

## Introduction

Current UK climate policies are not sufficient to meet UK climate targets (Committee on Climate Change 2019). Yet, in the past few years the issue of climate change has increasingly become a key concern for citizens in the UK as a result of the momentum built up after the 2018 report on *Global Warming of 1.5 °C* by the Intergovernmental Panel on Climate Change (IPCC), subsequent movements such as the Climate School Strikes and Extinction Rebellion (XR) (Rode 2019) and the growing number of climate emergency declarations worldwide. A global survey of public attitudes conducted in 2020 by UNDP showed that over 64% of people worldwide recognise that climate change is a global emergency (UNDP & University of Oxford 2021), and a UK survey conducted in October 2019 found that when asked what the UK’s most important issue to tackle in the next 20 years, climate change had become one of the two most frequently mentioned responses in comparison to being 13^th^ three years before. Additionally, the survey found that the UK government was assigned the main responsibility to prepare the country for climate change and mitigate it (Steentjes et al. 2020). This suggests that citizens are becoming increasingly concerned about climate change, with growing support for climate decision-making to be informed by public opinion (Sandover et al. 2021; Steentjes et al. 2021) and that there is a desire for the government to take the lead on tackling it, building up pressure for politicians and policymakers to adopt more stringent climate policies (Steentjes et al. 2020).

Citizen juries and assemblies on climate change are increasingly being run at every level of governance in the UK, from the first conducted in Camden (July 2019) to the national level UK Climate Assembly (January-May 2020). They were promoted since the wave of climate emergency declarations began in Bristol in 2018, and to date (February 2021) there have been over twenty climate-related mini-publics and other forms of participatory engagement on climate change in the UK (Howarth et al. 2021). It has been argued that these deliberative tools are a method for increasing citizen engagement, bridging the gap and building trust between the scientific, political and social consensus on climate change and increasing the democratic legitimacy of climate policies by creating more citizen-centred policymaking (Willis 2020; Kythreotis et al. 2019; Howarth et al. 2020; Smith and Wales 2000; Willis 2018; Devaney et al. 2020; Capstick et al. 2020). They are particularly important in the continuously evolving landscape of climate emergency declarations and resulting climate action plans requiring ‘new forms of democratic legitimacy’ which are increasingly concerned with embedding social justice and equity dimensions (Rode and Flynn 2020: 9). However, as citizen juries and assemblies on climate change only emerged in the UK in 2019, little research has been conducted into the extent to which these processes actually have an impact and promote broader citizen engagement on climate change policymaking. Thus, through a comparative case study of the Oxford Citizens’ Assembly on Climate Change and the Leeds Climate Change Citizens Jury, this paper investigates how these processes may differ in practice, the impact they are having on policy and how they are engaging the public into climate change debates.

## Literature review

### The case for more public participation on climate change policymaking

Many scholars have argued that increased public participation in climate policy formulation is required in order to help bridge the gap between the scientific and social consensus on climate change, increase authorities’ awareness of the wider public mandate for action on climate change and increase the legitimacy of climate policies (Willis 2020; Kythreotis et al. 2019; Howarth et al. 2020). Whilst politicians are aware of what protestors and activists think, they lack a clear sense of the wider public mandate for climate action and face constraints such as lobbying by powerful corporations and 5-year electoral cycles, preventing them from proposing the bold changes required to address climate change (Extinction Rebellion Citizens' Assemblies Working Group 2019; Willis 2019). Additionally, although climate change is a complex issue laden with scientific and technical detail, policy options for tackling it ultimately present complex (moral) choices among interests and values, making policy choices contentious along political, social, cultural and economic lines (Dietz and Stern 2008). However, the specific ways in which climate change should be tackled are still very contentious. The literature on deliberative democracy has highlighted processes such as citizens assemblies and juries as particularly appropriate to deal with very political and even moral considerations. It is argued that the deliberative element of these types of processes encourages listening to different points of view and seeking points of consensus, moving participants beyond the polarised nature of current politics (Dryzek 2005; Dryzek et al. 2019). In fact, citizen’s assemblies have already been used in the past for this purpose, with examples showing how they can largely reflect the views of the population as a whole (e.g. in the case of abortion legislation in Ireland) (Farrell et al. 2020). For example, the Irish Citizens’ Assembly (2016) considered abortion legislation, a very contentious and divisive issue. It recommended amending the Irish constitution to repeal the eighth amendment, which made abortion in Ireland illegal except for under specific circumstances. The Assembly’s recommendation was arguably instrumental in opening the door for a referendum on abortion, in which the Irish people accepted the recommendation of the Assembly and voted to allow it, with the outcomes being strikingly similar to that of the Assembly 2 years before (McKee 2018). Therefore, decisions should not be taken exclusively by experts but rather discussed and negotiated in the political arena, with both elected representatives and the public at large (Howarth et al. 2020).

Despite these challenges, until recently few attempts have been made to engage citizens or local areas in the need for, or benefits of, the transition to a zero-carbon, resilient society (Willis 2019). When policies are constructed without public engagement and support, the legitimacy of formal climate policy decisions may be undermined and there is a risk of public resistance. For example, the ‘gilets jaunes’ protests in 2019 emerged in France after the introduction of a fuel tax which was felt to disproportionately impact low-income households (Chrisafis 2018). Furthermore, there is evidence that when climate policy design does not take procedural justice into account (including proper participation options for affected communities), their outcomes are indeed unjust and socially negative (Lamb et al. 2020). Thus, many argue that citizens should be involved in shaping climate policy (Dietz and Stern 2008; Capstick et al. 2020; Niemeyer 2013; Kythreotis et al. 2019; Svara and Denhardt 2010).

### Citizens’ assemblies and juries: tools for including the public in decision-making on climate change

Interest in deliberative democratic processes has grown as a result of discontent with the confrontational and manipulative elements of current democratic practices and political discourses (Smith and Wales 2000). Their proponents argue that deliberative processes reduce polarisation of views on climate change (Ghimire et al, 2021) and improve the legitimacy and the quality of outcomes because, through meaningful deliberation, people engage in providing each other with compelling reasons to support or oppose particular proposals (Lafont 2017).

Citizen Assemblies and Juries, commonly called ‘mini-publics’, work very similarly. They are often commissioned, usually an institution with decision-making capacity (e.g. a local authority, national government or a regulatory body), and are composed of a randomly selected inclusive group of citizens, small enough to be genuinely deliberative. Citizen assemblies usually include 50–160 people, whereas citizen juries tend to include 12–30 people making them a smaller and therefore cheaper option (Bryant 2019; Roberts and Escobar 2015). An independent oversight panel or advisory board made up of key stakeholders is often set up to oversee the process (Wakeford et al. 2015). Participants receive expert information on a particular issue, cross-examine experts and deliberate with each other discussing different perspectives and trade-offs and come up with informed and considered recommendations of how to deal with that issue (Goodin and Dryzek 2006; Smith and Wales 2000; Roberts and Escobar 2015). The cost of running these processes can vary significantly (e.g. from £25,000 for small scale up to £500,000 for the UK Climate Assembly: Involve 2020a, b) depending on the number of participants, the facilitator commissioned to design and deliver, whether it is run online, incentives for participants, the extent of external public engagement and communication and the budget available (Shared Future 2019). The outcomes of these processes depend on the purpose they were commissioned for, how they were designed and delivered (see examples of diverse practices run locally in Brent, Newham, Camden, Leeds and Oxford: Bryant and Stone 2020).

Deliberative democracy tools can be used to engage with citizens by creating a structured dialogue between citizens, experts and politicians in order to chart a collaborative way forward on climate change (Howarth et al. 2020; Willis 2020). Carpini et al. (2004) argue that well-structured deliberation is expected to produce a number of positive democratic outcomes (e.g. enhancing faith in the democratic process, creating more informed political decision-making with public evidence and increasing the legitimacy of policies) which can help address several issues stalling stronger climate policy action, as well as making sure climate policies result in more just social outcomes.

The outcomes of these processes are shaped by the motivation for them being commissioned, the stage in the decision-making process at which they are commissioned and how they are designed and delivered in practice (Bryant and Stone 2020). Research into how the outputs of deliberative tools feed into traditional political structures and the influence they have on public policy decision-making is limited, and it is difficult to clearly identify the impact that citizen assemblies and juries have, as often the recommendations produced have an advisory role and compete with advice from other groups (Bryant and Hall 2017; Flinders 2015; Font and Smith 2013). Font and Smith (2013) claim that generally participatory processes only have a limited impact on final policies. Although there are instances where recommendations made by citizen juries and assemblies are referenced in and reflected in a council’s climate policy, it is difficult to prove whether such policies would have been put in place regardless (Bryant and Stone 2020). Thus, their impact on climate policy is by no means uniform and further research is needed to better ascertain in more detail the specific value they add.

The influence of citizens’ juries and assemblies on policy is complex with ways in which recommendations are seen as adopted varying: e.g. through an immediate response by the commissioning body, formal acceptance of recommendations by the authority, reference to a recommendation in a policy document/work programme, the actual implementation of the recommendation (Font and Smith 2013), or put for confirmation through a referendum. In addition, they can create a strong political platform for action by providing elected representatives with a political mandate on climate change which enables them to be more ambitious in their climate action (Bryant and Stone 2020).

### Contribution to broader public engagement on climate change

Citizen engagement involves collaborating and empowering citizens in decision-making, whereas citizen participation is often used to inform and consult with citizens to gain their views, assistance and support (Svara and Denhardt 2010). The level of citizen engagement can be assessed according to whether citizens have an opportunity to discuss ideas or efforts with other citizens and officials, whether engagement activities are citizen-centred, and whether decision-makers are willing to listen and take the results of the processes into decision-making (Svara and Denhardt 2010). Thus, although citizen juries and assemblies meet many of these criteria, the extent to which decision-makers take the results of the process into account varies, and this paper attempts to assess the impact these processes are having on decision-making in different contexts.

Citizen assemblies and juries on climate change can help to identify more nuanced and in-depth public opinions reached in a fair and informed way than is achieved by polling or surveys, testing and discussing a range of approaches to climate action and creating space for public engagement within policymaking by producing a set of prioritised recommendations (Bryant and Stone 2020). Therefore, they are a structured way of giving citizens the knowledge required on climate change and supporting them to collectively come up with policy recommendations which demonstrate the public mandate for climate action (Howarth et al. 2020). Additionally, they may increase momentum for action as a mandate put forward in a robust citizen assembly or jury process may be more difficult to dismiss than the ideas of left-leaning environmental campaigners (Bryant 2019).

If well publicised and acted on, citizen assemblies or juries may increase trust in government, a lack of which is a key issue in tackling climate change (Howarth et al. 2020; Willis 2020). Yet, many local authorities fail to ignite a wider public conversation around citizen assemblies and juries, and the potential for these processes to be a tool to start or enhance a wider public dialogue on climate change is often neglected (Bryant and Stone 2020). Public engagement beyond the formal process of the citizen jury or assembly such as through media coverage of process and follow-up events can contribute to engaging and communicating with the public more deeply on the outcomes of the process and can provide a means whereby decision-makers working on climate change can be held to the recommendations made (Delap 2001; Devaney et al. 2020; Capstick et al. 2020). For example, in France a large proportion of the population engaged with their national climate assembly through the media, generating a broader national debate, resulting in 70% of people in France having heard of the Assembly, and 62% supportive of the recommendations produced (Mellier and Wilson 2020).

The literature on deliberative democracy has widely discussed the relationship between mini-publics and the public at large (or maxi-public), particularly around questions of legitimacy and scale. There are a two main ways in which mini-publics have been justified as legitimate in their relation to the maxi-public: superior quality of the process leads to better decisions, and the representativeness (or inclusivity) of the participants leads to a outcomes that consider the views of the public at large (Lafont 2017; Brown 2018; Pow et al. 2020). Beyond these theoretical considerations, we consider that efforts to broaden the public debate around the issues tackled by citizens assemblies and juries (like the ones we mentioned above) are key for cementing their legitimacy and reach of their outcomes,

## Methodology

There is a need for research to compare and contrast different methods used in the growing number of national and local citizen assembly processes (Capstick et al. 2020), and to understand how citizen assemblies and juries on climate change are being used, as they may be used as a short-term consultation tool to give public legitimacy to political decisions which have already been made rather than to promote citizen engagement in policymaking and debates. Thus, the way in which climate change is framed in these processes and the topics discussed are important because they could influence the conclusions and recommendations reached (Wakeford et al 2015). This study aims to address this by comparing the Oxford Citizen Assembly on Climate Change and the Leeds Climate Change Citizen Jury, both conducted in 2019 in the UK, to investigate the impact that citizen assemblies and juries on climate change are having on citizen engagement and climate policymaking in UK cities.

### Methodological approach

A qualitative research design was adopted to gain understanding of key actors’ perspectives and experiences from their involvement in the processes and climate policymaking in the cities more generally, through rich and detailed data gathered during interviews, going beyond official reports and policy statements (Seale et al. 2011). A purposive sampling approach was adopted where those involved in running the processes in Oxford and Leeds directly including City Councillors, policymakers, facilitators and members of the oversight and advisory group, or those involved in climate policymaking in the city councils more generally were approached for interviews. Thereafter, a snowballing approach was used by asking interviewees for suggestions in order to reach others who were involved but not mentioned in public reports; a total of thirteen individuals were interviewed (Table 1). A semi-structured interview technique was adopted as this allowed the study’s aims to be investigated by analysing common patterns and themes in interviews through the use of an interview guide whilst maintaining scope for unanticipated themes to be further explored by asking unplanned questions, allowing for more thorough analysis (Howarth et al. 2018; Braun and Clarke 2018, p. 78). As the interviewees were volunteers, the participation of whom was subject to their availability in the time period that the research was completed, the participants in both cities represented a varied sample from all constituencies and/or political parties (for the purposes of maintaining participant anonymity, we have excluded information on party affiliation). The focused representation of the interviewees may be seen as a limitation to the study; however, these individuals presented information-rich cases in relation to the research aims (Baxter and Eyles 1997; Braun and Clarke 2018). Whilst we acknowledge that the values, biases and agendas of the participants may influence their responses, interviewees spoke in their professional capacity specifically in relation to the Leeds and Oxford case studies. This provided a fairly good representation of knowledge of these processes and their subsequent role in influencing policymaking in the cities, with many participants directly involved in climate-related policymaking, without necessarily being representative of broader constituencies.

**Table 1:** Table of participants, coded

Code	Meaning	Number of interviewees
LGO	Local Government, Oxford	4 (LGO1, LGO2, LGO3, LGO4)
LGL	Local Government, Leeds	4 (LGL1, LGL2, LGL3, LGL4)
PO	Practitioner for Oxford Citizens’ Assembly	1 (PO)
PL	Practitioner for Leeds Citizens’ Jury	1 (PL)
OO	Involved in running the Oxford Assembly	2 (OO1, OO2)
OL	Involved in running the Leeds Jury	1 (OL)
		Total: 13

For the purpose of anonymity and confidentiality, all names and positions of participants were coded. Interviews were audio-recorded with the participants’ consent and were transcribed by the researcher, and a thematic analysis of transcripts was carried out using NVivo. The following themes were covered in the interviews: (i) reasons why interviewees thought a citizens jury or assembly on climate change was used; (ii) thoughts on the structure of the citizens’ jury or assembly; (iii) thoughts on the recommendations produced; and (iv) how influential interviewees thought the process has been in terms of climate policy and citizen engagement in the city. The research adopted a grounded approach, where nodes were built up whilst analysing interviews in order to identify common themes. The nodes were then reviewed, refined and collapsed into primary and secondary nodes to provide more depth of analysis (Fuji 2018; Rapley 2011). Once the nodes were finalised, the transcripts were reviewed to ensure coding was consistent.

### Case studies

The **Oxford Citizens Assembly on Climate Change** was run as part of the Council’s response to their Climate Emergency Declaration in January 2019. The process was commissioned by Oxford City Council and facilitated by Ipsos MORI, and an advisory group including key stakeholders was set up to provide governance and oversight. The Assembly attempted to answer the question: ‘*The UK has legislation to reach ‘net zero’ by 2050. Should Oxford be more proactive and seek to achieve ‘net zero’ sooner than 2050?*’.

Five themes which the council felt it had control and influence over—Waste Reduction, Buildings, Transport, Biodiversity and Offsetting and Renewable Energy—were selected to be covered in the process. Within each theme, Assembly members were then presented with three possible futures for Oxford related to how ambitious climate action would be in reaching ‘net zero’. Forty-two residents were randomly selected as a representative sample of the city to participate, and the process ran over two weekends from September to October 2019. The resulting recommendations favoured stronger climate action, with the majority of participants voting for the most ambitious scenario in each theme covered (Ipsos MORI 2019). The process was shared with the public through the Council’s social media accounts, and videos of the presentations and assembly member questions are available on their YouTube channel.

The Assembly was well received by the Oxford City Council, who responded in December 2019 by setting a Climate Emergency Budget committing over £1 million additional funding and £18 million of capital investment to address the climate emergency. The council further committed itself to a range of actions, such as ensuring the organisation was net-zero by 2020, which has now been pushed back to the end of 2021, establishing a Zero Carbon Oxford Partnership, creating new carbon budgets and more (Oxford City Council 2019a, b,  2020a, b, c, d). The Council also outlined its current and future plans to reach net zero in each area covered in the Assembly and claimed in its Cabinet report that the Assembly had informed the Council’s plans to be more ambitious in achieving net zero (Tullar and Colwell 2019). The council further claimed that the Assembly would feed into its Sustainability Strategy in early 2020, although this is yet to be published (Oxford City Council 2019a, b,  2020a, b, c, d). Nevertheless, the Council have published their 4^th^ Carbon Management Plan for 2021/22–2029/30, which outlines how they will become a zero carbon council by 2030 (Oxford City Council 2021). The Council also held a youth climate summit in November 2020, which was part of their response to the Assembly (Oxford City Council  2020a, b, c). Thus, this suggests that the Assembly is influencing Oxford City Council’s climate policies.

The **Leeds Climate Change Citizens Jury** was run in 2019 as part of the Big Leeds Climate Conversation which began after Leeds City Council declared a Climate Emergency and was run by the Leeds City Council and the Leeds Climate Commission. The Jury was commissioned and funded by the Leeds Climate Commission. The Jury was to guide the future work of the Commission, and Leeds City Council agreed to formally respond to the recommendations. The Jury was facilitated by professionals from Shared Future and an oversight group made up of key stakeholders oversaw the process. The key question presented to the Jury was: ‘*What should Leeds do about the emergency of climate change?*’. Twenty-one residents were recruited as an inclusive sample of the city, and the Jury was run through eight evening sessions. The Jurors chose four topics which they wanted to focus on in more detail during the process: transport, communication and community involvement, housing and finance. Twelve recommendations were made on a broad range of issues and were ordered in terms of how many votes they received, and members also requested progress reports every 3 months for a year after the Jury ended (Shared Future 2019). The process was shared with the general public through live streaming the Jury’s launch event in which the findings were presented. Videos of presentations and interviews with the commentators are publicly available on the Big Leeds Climate Conversation YouTube channel and the Leeds Climate Commission social media accounts.

The Big Leeds Climate Conversation, as a whole, influenced the January 2020 Council Climate Emergency Update Report (Cook and Evans 2020a, b) which contained a brief description of the Citizens’ Jury and claimed that its recommendations would be presented to the Council’s Climate Emergency Advisory Committee (CEAC). The report also referenced the recommendation on the use of green bonds and crowd-funding when discussing a project the Council was running to explore the use of crowd-funding to finance PV systems on Council buildings. The March 2020 CEAC response to the Jury welcomed its contribution, claimed support for the recommendations and outlined the council’s plans in regard to the areas they covered (Cook and Evans 2020a, b). As we illustrate further in the findings section below, it appears that, although the Council supported the recommendations, the Leeds process, in comparison to the Oxford process, did not lead directly to new climate-related policies.

## Findings

In this section, we present findings from some key issues relating to recruitment and framing, the purpose, structure and impact of these processes, engagement with the wider public and funding, all of which have a bearing on how far these mini publics increased engagement with climate change and led to more citizen centric policymaking.

### Recruitment and framing of topics

The two processes differed in many key ways. In terms of the recruitment methods, in Oxford, the majority of participants were recruited from a pre-existing Oxford City Council Citizens’ Panel. The sample was topped up where there were gaps in the Assembly’s demographic composition through targeted on-street recruitment to reach required, typically under-represented, groups (Ipsos MORI 2019). Although everyone in Oxford’s population had an equal chance of being recruited to the Citizens’ Panel, the majority of participants in the Assembly were recruited from this pre-existing group. In contrast, in Leeds the Oversight Panel agreed the profile of jurors to reflect local diversity prior to recruitment. Participants were selected based on agreed profiles, with factors such as level of deprivation and attitudes to climate change. Additional members from marginalised groups were also recruited, to ensure their voices were not drowned out during the Assembly, as they often are in climate debates (Shared Future 2019). Therefore, in both cases the demographic profile of the participants was taken into account in an attempt to ensure that samples reflected the diversity of both city’s populations. However, in Leeds the participants’ attitudes towards climate change was also a key factor, ensuring that participants with a range of attitudes took part. Although participants’ attitudes towards climate change were monitored in Oxford, it was not used as a selection criteria for recruitment (Ipsos MORI 2019).

Further, the Oxford Citizens’ Assembly was run directly by the City Council whereas the Leeds Citizens’ Jury was run by the Leeds Climate Commission (and endorsed by the City Council). The framing of the question in Oxford narrowly focused on the net-zero goal and whether the city should be more ambitious, whereas, in Leeds the question was broader, focusing on the city’s overall response to the climate emergency. The decision to pre-select the themes and options for the recommendations in Oxford and focus on those that the Council could control or influence meant that, although participants had little say in what was covered, the outcomes of the process were more focused on the key areas the council wanted to engage with citizens on. In contrast, in Leeds the participants choosing the themes to be covered meant that the process was more citizen-led.

### Purpose of the process

The key reasons for using the Citizens’ Jury and Assembly processes were broadly aligned in both Oxford and Leeds. The imperative to act after declaring a Climate Emergency, whilst bringing citizens on board, was a strong driver in both cases (LGL3–4, LGO2–3). Interviewees highlighted a desire to increase citizen engagement on climate change to encourage behavioural changes (LGL1–2, LGL4, LGO3), involve citizens in climate policymaking rather than imposing policies on them (OL, LGL2, LGL4, OO1) and better educate citizens on climate change (OL, LGL2, LGO1). In Leeds, interviewees saw the value of gathering deliberated opinions from citizens from diverse backgrounds, giving them an opportunity to gain an in-depth understanding of how policies will affect different people (PL, LGL2–3). Furthermore, in both cities the processes were seen as an opportunity to engage with the public and assess views on more ambitious climate change policies ( LGO3, LGL2–4, PO).

*“I think the principal thing for our citizens’ assembly was, in Oxford was, how ambitious do these people want us to be, and how ambitious do they want the councillors to be.”* (LGO3)

Thus, our evidence suggests that these processes were used in order to increase informed citizen engagement in climate issues and policymaking, and to gather informed and diverse public opinions on climate action. The purpose of these processes was not to directly devolve decision-making power to participants, but rather to explore and integrate these deliberative democratic practices within existing institutional arrangements and representative democracy structures.

### Structure of process

The most significant difference between the Oxford and Leeds processes in terms of structure was the choice of question and topics. In general, interviewees from Oxford were positive about the decision to limit the number of topics to be covered beforehand, indicating that the Assembly focused on areas that the council felt they could control or influence (LGO2–3) and did not want to overwhelm participants with too much information (LGO2). Indeed, such a tight framing of the themes can allow the final recommendations to deliver clear messages to policymakers on what actions to take (Bryant and Stone 2020). However, an assembly with a pre-determined structure may be performing more of a consultative role rather than genuine citizen engagement as they are choosing from a list of pre-prepared strategy options, denying citizens the opportunity to present their own solutions to issues (Bryant and Stone 2020).

‘*Based on that evidence base of ok where is our biggest emitting sectors we do know that’s where we need to focus and on top of that, yeah lots of discussion with various sort of experts in the field of what… was important to include’* (LGO4)

Yet, there were some suggestions that despite limiting the Assembly to cover five themes, the scope had still been too broad given the amount of time it had and its task to assess both a net-zero target date and the participants views of the actions required in each level of ambition within the themes (OO1–2, LGO1).

*‘I do think it was too many in that there wasn’t sufficient time for people to be given to think through the complexities and what they thought about them in specific cases.’* (OO2).

In Leeds, participants selected the topics discussed in the process, focusing on those they felt were important to tackling climate change in Leeds, making the process more citizen-led (PL) insofar as participants were able to select which topics to focus on in the Assembly and the recommendations that were produced. The Jury was on the climate emergency in general and run by the Leeds Climate Commission, allowing for a broader set up (LGL4) and citizen steering at different stages. Furthermore, the broad structure of the Jury allowed participants to come up with recommendations which were not necessarily on the Council’s or Climate Commission’s agenda, such as that which recommended stopping the Leeds Bradford airport expansion (PL). This broad structure arguably allowed the jury to challenge existing policy, an important feature where policy-decisions (such as allowing an airport expansion) could negate all other efforts to combat climate change in the city. This suggests that creating scope for participants to steer the processes in terms of the topics covered within the jury or assembly and the recommendations produced is important when covering complex and controversial topics such as climate change (PL). However, there were suggestions that the scope covered in the Jury had been too broad (LGL2, LGL4).

*‘That would be one of my reflections that it’s just too broad a topic, you know they had something like 30 hours I think but even so, you know, 30 hours talking about the climate emergency and public transport and housing and financial markets, you know you can't go into depth on anything.’* (LGL4)

Overall, this suggests that the structure of the citizen assembly or jury has a significant impact on the type of citizen engagement achieved in these processes, as it determines whether the recommendations produced are citizen-led or reflect a more consultative process of citizen participation.

### Impact of the process

In both cases, the Councils responded to each recommendation with reference to their existing or planned actions in those areas and reference them in some reports as supporting initiatives which are in line with the recommendations (OL, LGL3), both of which according to Font and Smith (2013) suggests that they are having some influence. We can make a distinction between these processes having a direct impact (e.g. recommendations get directly turned into policy) or an indirect impact (e.g. influencing policymakers and participant’s views on climate change and climate action).

#### Direct impacts

The direct impact of the recommendations produced through the assembly and jury varied. In Leeds, interviewees indicated that the recommendations were too broad or vague to provide useful insights for specific and complex policy issues such as improving energy efficiency in housing (LGL2) or citizen engagement with and communication of climate change to the public (LGL4). This supports the argument by Font and Smith (2013) that vague recommendations may not offer useful guidance to policymakers, suggesting that if a citizen jury or assembly is being used to inform policymaking on specific issues rather than gage broader public opinion it may be more useful to run them on narrower topics so they can be studied in depth and produce focused and practical outputs.

*‘In my view, the recommendations need to be SMART. Specific, measurable, achievable, realistic and time-bound. Well, specific, I don’t think they’re as specific as they could have been…’* (LGL1).

Nevertheless, in Leeds, the recommendations aligned with the Council’s plans to tackle climate change and interviewees claimed it endorsed pre-existing policies or initiatives (LGL2–4). Yet, the jury was part of a wider process of citizen engagement, the Big Leeds Climate Conversation (LGL4), which makes its impact on climate policymaking more difficult to identify, and was run by the Leeds Climate Commission rather than the council itself which may have influenced the extent to which the council felt it had to act in response to the Jury.

*‘The results from the citizens’ jury aligned very very closely with what the, the council, you know, was intending to do anyway, so it was a kind of a, kind of an endorsement verification process.’* (LGL3)

In Oxford, interviewees highlighted that the Assembly provided support for the introduction of a policy package including £19 million additional funding on climate change, which one interviewee claimed had been drafted and approved beforehand but was presented as a response to the assembly (LGO1, OO1, PO). Yet, it was suggested that Oxford City Council’s response to the recommendations had been delayed by the COVID-19 pandemic, implying that it could have more of an impact in the long-term (OO2). Nevertheless, one interviewee claimed that the Assembly supported pre-planned policies rather than changing the councils’ approach, which suggests that the Assembly did not change the council’s policies although this may be expected given the Assembly was framed according to the councils’ climate policy options (LGO1, LGO3).

*‘…it was all things they were going to do anyway. Nothing changed in the local plan for instance, so the local plan that we’ve just adopted will not see all new buildings being zero carbon. They could’ve put it in but they didn’t.*’ (LGO1)

*‘I don’t think we would have done what we did in the time-scale that we did without that process… it’s really helped put it on the agenda and as an organisation it’s completely flipped what our corporate policies are… zero carbon became one of our primary corporate priorities and for the first time as well it meant the whole organisation had to be engaged.’* (LGO4)

The lengthy process of writing, approving and implementing policy was also referenced, suggesting that the influence of the citizen jury and assembly recommendations may be seen in years to come, but also that policies that are put into place directly after these processes are run were pre-existing, meaning they are not necessarily a direct consequence of the processes (LGO1, LGL1). However, in both cities there was mention of how the recommendations were not intended to directly make policy decisions, and some interviewees expressed doubt that the recommendations would have a direct impact on policy (LGL3–4, OO2, LGO1, LGL1). This implies that citizen juries and assemblies on climate change are playing an advisory role, providing legitimacy for and momentum to launch pre-planned policies at a faster pace rather than truly empowering citizens in decision-making (Wakeford et al. 2015). If these processes are to represent citizen engagement on climate change rather than citizen participation, it should be clear from the beginning how the process will inform decision-making, as some interviewees implied that they were unsure what impact the processes had had or why it was used (LGL1, LGO1, LGL4; Goodin 2008).

The influence of the recommendations may reflect the stage in decision-making in which they were used, as Roberts and Escobar (2015) claimed that citizen assemblies are most influential if done at the agenda-setting stage of the policy process, rather than when policy approaches had been drafted as was the case in Oxford where participants chose between a narrower set of options to form their recommendations. Yet, climate policymaking is ongoing, and the processes still may play an agenda-setting role by indicating the level of ambition the council should strive for (Roberts and Escobar 2015).

#### Indirect impacts

The value of the processes in creating momentum and giving politicians and policymakers greater confidence to pursue stronger measures on climate change was highlighted in Leeds and Oxford. In Leeds, the Jury created momentum and gave officials backing for stronger measures by increasing their confidence that the public would be supportive of them (LGL2, OL). Similarly, in Oxford interviewees highlighted how the Assembly created momentum by pushing the Council to be more ambitious in their climate action and reassuring politicians of the public’s support (LGO2–4, OO2–1, LGO1). This supports the argument that the biggest impact these processes have is to create a strong political platform for action by providing elected representatives with a public mandate on climate change (Bryant and Stone 2020). Yet, the need for the momentum to be sustained in the long term by continued reference to the recommendations by other actors in the city was highlighted in Leeds (OL).

*‘It gave the political leadership…the political momentum to push through more funding… for low carbon initiatives.’* (OO1).

*‘It certainly added weight and credibility and helped with momentum, would it, would it completely change the council's views on something, probably not’* (LGL2).

Furthermore, the citizen assembly and jury processes were valued as tools to help overcome some of the key challenges climate action faces. In Oxford, the Citizens’ Assembly was seen as a response to and an opportunity to overcome the struggle within the political system to effectively tackle climate change (PO). Reasons for this included short political cycles clashing with the long-term issue of climate change (LGO3), the lack of trust in governments and influence of lobbyists such as the fossil fuel industry (OO1) and the failure of elections in informing government of public opinions on climate change as votes generally reflect people’s short-term interests (LGO1). This was also suggested in Leeds, where the citizens’ jury was described as an alternative and deliberative form of democracy outside of the normal realm and challenges of local government (LGL3–4).

*‘If you just leave it to the ballot box, it’s hopeless. I mean our democracy is fundamentally broken, it doesn’t tell you what people think about climate change because they’re more interested in what bus routes are running and things like that, it’s short-term stuff.’ (LGO1)*

*‘Politicians are absolutely driven by the political cycle…and a lot driven by what is in the public domain and in the press, so they…really do react to that kind of reputational arena, and you and I both know that sustainability is a long-term issue… and those aren’t great in a 4 or 5 year political time-scale, and we’ve got a kind of clash between a long-term and quite potentially ‘unsexy’ area of work, against political priorities, press, the need for new and news….’* (LGO3)

Furthermore, in both cities the processes demonstrated that citizens who are educated on climate change and the various issues surrounding it through the right communications are more likely to support and engage in stronger climate action (LGL2, OL, LGO2). Additionally, interviewees highlighted that better educating and communicating with citizens on climate change were recommended in both processes. Thus, the processes seem to have encouraged the councils and other parties to increase and improve future citizen engagement on climate change more broadly (LGO2–4). However, in Leeds it was suggested that the jury could have provided more in-depth insights into how the participants thought communications could be improved to engage the public in climate change debates, highlighting the desire for more practical recommendations (LGL4).

*‘Part of what came out of it was a huge engagement programme and actually some of that has survived… we were due to do workshops and all sorts… that was not something we were planning to do before this process so, yeah huge engagement programme now lined up.’* (LGO4)

Therefore, the indirect impact of deliberative democratic processes can be significant and positive. Participants themselves find the experience enriching, and politicians and policymakers can find it emboldening and reassuring in their chosen policy path, balancing some of the other influences they are usually subject to (e.g. the media, lobbying groups, electoral cycles).

It is important to critically analyse how these processes are being run and the impact they have in practice. Direct impacts or indirect impacts may not be immediately apparent, which led to some interviewees raising concerns that the impacts of these processes were not being critically assessed due to the heightened attention on climate change action (LGL4, LGL1).

*‘I looked at the recommendations, I think it was Camden because there weren’t that many before ours, and I read the recommendations and I thought exactly the same as I thought about ours, that it didn’t tell me anything I didn’t already know. But if you were to listen to the politicians at Camden speaking, well it’s the best thing that’s ever happened, you know it’s really changed the world, you know I struggle to see that, but I think it’s a bit like the emperor’s new clave, if you know what I mean by that, that you’re almost on the wrong side of life if you’re not saying they’re great, because everybody’s just singing and dancing,’* (LGL4).

The role that citizen assemblies and juries on climate change were seen to have within the wider democratic policy making process was unclear. There was a feeling that if these types of processes were to have a direct impact on policies it would be undemocratic (LGL2). Again, this represents a tension of how to embed deliberative democratic practices that involve the public directly within institutional arrangements based on representative democracy, a tension between giving some power back directly to a group of citizens, potentially at the expense of elected councillors.

*‘There was an implication by a few people… it was almost like we should leave it to the jury and just do what the jury says… well that’s not a position that a democratic body would take, it wouldn’t actually say you know, we have been elected by the electorate but we are actually going to in some senses abdicate responsibility and allow a jury to determine policy, it’s got to be, it’s a form of, informed consultation, and kind of consultation has a weight obviously.’* (LGL2)

### Engagement with the wider public

There was agreement in both cities that the citizens’ jury and assembly needed to better draw the wider public into debates on climate change. The value of the Leeds Jury in relation to its cost was questioned due to its perceived failure to influence or draw a significant proportion of the wider population into climate change debates (LGL1, LGL3–4). This implies that in order for these processes to be seen as worthwhile they need to engage the wider public in climate debates. Similarly, the failure to energise and involve the wider public through the process of organising the Oxford Assembly was criticised (OO1, LGO1). This suggests that citizen juries and assemblies on climate change present an opportunity to engage with the wider public and build trust, an opportunity which was missed in these two instances. Better media coverage of citizens’ juries and assemblies on climate may engage and communicate with the public more deeply on the debates had in and generated by these processes (Goodin 2008; Delap 2001; Devaney et al. 2020; Capstick et al. 2020). Thus, broader citizen engagement should be a key focus for future climate citizen juries and assemblies for them to achieve their engagement potential and be worthwhile processes.

*‘Ultimately you know 25 people across 800,000, they might each go back and talk to 10 people but that still only reaches 250. So I think there’s something about trying to work out how you get more out of that legacy, and how they become more kind of influential (…) it can be quite a limited role if that kind of legacy bit is not properly planned out and talked through.’* (LGL4)

*‘The very process of organising a citizens’ assembly should energise and involve in it, you know lots of people, and that wasn’t really achieved.’* (OO1)

Interviewees suggested that citizen assemblies and juries could be used again in the future with a narrower scope or at more local scales (OO1–2, LGL4). Before the COVID-19 pandemic, there was discussion in Leeds of having citizens’ juries at a regional-level on adaptation (LGL3) and in the city on transport as this was highlighted as being a particularly contentious and difficult issue (LGL2–4). This suggests that policymakers saw these as being useful processes for engaging with citizens on contentious climate-related issues.

*‘I don’t think just having more and more citizens assemblies in the same place on, on climate change is, is the way forward (…) actually, you probably need to go to the next level of granularity and ask questions like, well, what, what should Oxford’s transport system look like?’* (OO2)

### Value for money

Citizen juries and assemblies were frequently referred to as expensive processes, especially in Leeds where funding primarily came from research grants (LGL3–4, OL, LGO1, LGO3–4). The price of the Oxford Assembly process was estimated at £200,000–£250,000 (LGO1, OO1) and the Leeds Jury at £30,000–£40,000 (LGL3, LGL4, OL). Consequently, there was concern over how to make them more accessible as most local authorities or organisations lack funding to run these processes, especially given the wider context of the pandemic, or prioritise delivering projects rather than extensive public consultations (OL, LGL3). The use of other deliberative tools on narrower topics was suggested as an alternative way to engage with citizens on climate change (PL, OO2). Focus groups were identified as being as good or better tools to gage public opinions on specific issues due to the in-depth discussions and practical insights they can provide and the lower cost of running them (LGL3–4, LGO4). Moreover, other methods of informing the public were suggested as being better value for money, such as a radio campaign or educational programme in schools which may reach more people, further emphasising the need to involve the wider public in these processes if they are to be worthwhile (LGL4).

*‘I think reflecting on the amount of staff that was involved and the money it cost to run it, you have to reflect on whether there are more effective ways, or at the end of the day this is what it comes down to whether there are cheaper ways to achieve something similar.’* (LGO4)

*‘Has it raised greater awareness, maybe but there’s no kind of evidence to show that, in which case you know I think our kind of assessment up front was that it was a lot of money to talk to 25 people even though they come away as really good advocates, unless they’re advocates that go out and talk to 1,000 people or you know, from a value for money perspective you know it’s not the best value for money.’ (LGL4)*

Yet, the value of having an inclusive sample participating in a jury or assembly was highlighted, suggesting that in-depth citizen engagement with a representative sample at more affordable rates was desired (LGL4). However, this is difficult to achieve as recruiting representative samples is a resource-intensive process (OL, LGO4). Therefore, the price of running and the small number of the target population which they engage seems to be key challenges to these processes.

*‘You know those 24 people in Leeds they came up with recommendations which were broadly consistent with what we were going to be doing anyway, so that, that, that’s interesting, that’s an interesting thing, but given that that’s the fact then could we use that 30,000 pounds maybe to try and get carbon literacy in I don’t know, the 5 largest employers in the city and maybe reach, I don’t know, 20,000 people’* (LGL3).

## Discussion

The two case studies have allowed us to draw some important links between the topics discussed in Sect. 2: meaningfulness of participation, legitimacy and effectiveness. In this section, we will analyse each of these in turn.

The meaningfulness of participation relates to how the inputs from participants were conceived, both before and after the processes. The interviewees primarily saw these processes as a way to increase engagement, educate participants and learn about different impacts of policies they were considering or may have overlooked. Therefore, it is not surprising that many interviewees did not report any direct impacts from the processes, at least not for now. Some interviewees also failed to identify any indirect impacts and implied that the link to decision-making was unclear. Some even suggested that these processes could have been replaced by focus groups or increased climate/carbon literacy suggesting a doubt in the value of the process. If participation is not considered in a meaningful way from the beginning, it is difficult for it to become meaningful down the line. Therefore, both the direct and indirect impacts should be clear to participants and commissioning bodies from the beginning of these processes.

The thread of legitimacy linked aspects of the purpose, structure and impacts of the processes. In terms of structure, the Oxford Citizen Assembly on Climate Change and the Leeds Climate Change Citizen Jury differed in the extent to which they truly engaged with the public. In Oxford, the decision to pre-set the topics discussed and the shape of the recommendations created more of a consultative process of citizen participation than citizen engagement as the process was not necessarily citizen-led. In contrast, in Leeds the jurors decided on the topics to be discussed and wrote the recommendations themselves, suggesting that this structure better represents genuine citizen engagement on climate change as a whole. However, this is not to suggest that the structure of the processes in these two cases are indicative of two types of Citizen Juries and Assemblies, but rather that these two cases demonstrate how different they can be in practice, which should be analysed when considering their legitimacy and influence over climate action and policymaking.

Questions of who held power to design policies, a small group of citizens or elected representatives, were highlighted. On the one hand, it was interesting to see how there was some reluctance to allow the mini-publics to directly design (e.g. Oxford with pre-determined policy options) or influence the design of specific policies (e.g. Leeds where so far the recommendations have not resulted in specific policies). On the other, referring to responsibility and accountability, the jurors or assembly members had no official or institutional responsibility^1^ and thus no accountability, to the rest of the citizenry (Fuller 2019). Furthermore, mini-publics themselves and their outcomes are not immune to the influence of powerful and vested interests. There is always a possibility that lobbying and corruption could influence participants during the process (and thus the outcomes) or the way in which the outcomes are acted upon. We are not aware of any evidence of the former, whilst the latter is certainly conceivable as the outcomes of mini-publics work their way through existing political institutions.

Finally, the thread of effectiveness also surfaced in the impact and engagement with the public themes. Two elements are key: the indirect impacts and the stage in the decision-making process at which these processes were undertaken. In relation to the latter, mini-publics at the agenda setting stage might avoid some issues of legitimacy (because they do not lead to specific policies), as well as being more effective in steering policymaking in a certain direction and providing more meaningful participation to those involved. Then the issue of the trade-off between breadth and depth, and thus vague or specific recommendations, becomes clearer, because mini-publics would deliberate on broader directions and priorities, whilst specific policies could be developed at a later stage by elected representatives whilst including citizens more broadly. In relation to the former, we consider the indirect impacts to be particularly important. The effect on policymakers can be vital, providing a way of re-balancing the sources of influence they are subjected to, giving a group of inclusive citizens a louder voice. In both cities the value of the processes in creating more momentum to act on climate change was highlighted, enabling an increase in the pace of climate action, especially in Oxford.

However, the failure to engage with the wider public on climate change through the processes was highlighted as a missed opportunity, whilst the value for money of the processes considering the small number of participants which were engaged was questioned. Thus, although citizen juries and assemblies have the potential to engage with the wider public, they have not always done so. In order for these processes to be seen as worthwhile given their high cost, they should aim to engage the wider public in debates during the processes through large-scale communication campaigns for example, although this would likely increase their cost further. Moreover, we should remember that people ‘participate’ on climate policy issues through a vast mosaic of ways, what Chilvers et al. (2021) have called ‘ecologies of participation’, including everyday practices, community activism and organising, campaigning, etc. Thus, as well as communicating what is happening during deliberative processes, we should strive to find ways to listen and take into account more informal ways of participation.

The impact of the COVID-19 pandemic on the outcomes of these processes and climate action have been significant. Interviewees claimed that the increased funding which had been allocated to climate change in Oxford City Council’s budget has disappeared due to the large financial impact of the pandemic, suggesting that the pandemic is slowing down and perhaps even preventing city-level climate action. In Leeds, a citizens’ jury specifically on transport was being considered, yet due to the financial impact of the pandemic it is unlikely the council will be able to fund it. Similarly, in Oxford the pandemic has disrupted plans to engage further with the public. However, in both cities it seemed that the momentum behind climate action had not disappeared and that climate change was still a priority. Nevertheless, the economic impact of the COVID-19 pandemic on councils may affect their ability to run citizen juries and assemblies on climate change in the future, and may stall climate action.

## Conclusion

Citizen assemblies and juries on climate change are increasingly being used in the UK as a mechanism for engaging the public on the issue and providing them with a role in informing climate action. It is however challenging to fully understand the real impact of these processes given they are such a recent phenomenon.

This paper suggests that the direct impact of citizen juries and assemblies on climate change policy making is not uniform and depends on the stage at which they are conducted, who commissions them, their structure and how meaningfully citizen participation is designed. However, their indirect impact can be significant, especially by balancing the sources of influence that policymakers are subject to and increasing political momentum for further and faster climate action by councils. Specific indicators of their impact, whilst to be considered with a degree of caution given the recent nature of these processes, could, for example, include mentions by policymakers and policy documents although this cannot necessarily be considered an indicator of uptake and influence. In future, indicators of impact could include direct/indirect reference/use in local decision-making processes on climate change (e.g. expansion of Leeds Bradford Airport), participants of citizen assemblies or juries becoming embedded in further climate governance (e.g. a selection of Jurors of the Croydon Citizens' Assembly on Climate Change were invited to sit as Commissioners on the Croydon Climate Crisis Commission in 2020) and demonstrable increase in public engagement and awareness on climate change (e.g. Big Leeds Climate Conversation).

However, it seems there is a missed opportunity in these processes to engage the wider public in debates around climate change and how it should be tackled. This suggests that future citizen juries and assemblies should seek to utilise the momentum they generate by not only to engage the participants in climate change debates but also to engage the wider public to improve legitimacy and promote learning and engagement (see Sandover et al. 2021 for a discussion of this in the context of the Devon Net Zero Citizen Assembly). Nevertheless, both processes analysed in this paper recommended more communication with the public on climate change which both councils seem to have taken on board, implying that citizen assemblies and juries on climate change may encourage councils to increase their work to engage citizens in the debates around climate change.

The findings of this research suggest there is a scope for future research on citizen juries and assemblies on climate change. Long-term research on the impact that these processes have on climate policy could assess whether these processes are truly influencing long-term climate policymaking, as ultimately their future impact may not be realised for some years. Further, this paper focused on two of many citizen assemblies or juries which have/are being run on climate change in the UK and beyond. Although this research found differing structures being used in these processes, it is beyond the scope of this paper to determine whether these cases represent two typologies of citizen assemblies and juries. Thus, future research could study a wider range of these processes to create typologies to better distinguish them and critically assess them. Core elements of such a typology could include whether the process is bottom-up or top-down, the stage in the decision-making process it was run, the length of the process, whether they meet generally accepted standards and the commitment made by the commissioning body to act on its recommendations.

## Data Availability

Data is freely available on request.

## Footnotes

1 It should be pointed out that, we know anecdotally, many participants take their role very seriously and with a great sense of responsibility as part of their civic duty (see Jacquet 2019).
